# How to review a paper on medical education

**DOI:** 10.15694/mep.2019.000158.1

**Published:** 2019-07-29

**Authors:** Richard Hays, Barbara Jennings, Trevor Gibbs

**Affiliations:** 1 James Cook University and University of Tasmania; 2 University of East Anglia; 3 Association of Medical Education in Europe

**Keywords:** medical education, publishing, guidance for reviewers.

## Abstract

This article was migrated. The article was marked as recommended.

There has been a substantial increase in the number of medical and health professional education manuscripts being submitted to an increasing number of journals in this field. More reviews and more reviewers are needed to facilitate discussion of both relevance and quality of those manuscripts. MedEdPublish relies on readers and Review Panel members to contribute to this process, thereby helping to maintain standards in medical and health professional education publishing. This article provides guidance that is most relevant to reviewers and potential authors for MedEdPublish, but may be relevant to publishing in other medical and health professional journals.

## Introduction

One of the major challenges facing academic publishing is that manuscript reviews are increasingly difficult to source. Although the number of academic journals in medical education has increased over the last 15 years or so, the number of manuscripts submitted has increased even more. Accordingly, the demand for peer review of manuscripts has also increased dramatically. In response, many journals now reject a substantial proportion of manuscripts without the benefit of peer review. Anecdotally, editors often invite 6-7 reviewers to receive 1-2 reviews. Those known to do reviews are being asked more frequently. For example, these authors often receive at least one review request each day, which is very difficult to fit into busy lives.

This situation threatens one of the underlying principles of academic endeavor because peer review is regarded as the most effective way to maintain academic standards. While MedEdPublish is a post-publication peer review online journal, with a high proportion of submitted manuscripts published relatively rapidly without the potential for some causes of publication bias ( Hays, 2016), the journal depends upon reviews of published articles from both our Review Panel members and the general readership. The discussion threads that follow amongst our community of practice maintain both standards and relevance. More reviewers and more reviews are needed to allow the journal to continue to meet its objectives.

This article aims to assist two audiences. The first is the members of our Review Panel, because feedback has suggested that some reviewers feel uncomfortable attaching their names to open reviews that may be critical. The second audience is the general readership, because MedEdPublish genuinely wants more readers to write reviews and contribute to discussions about ideas and research findings. Writing reviews may also be a valuable learning experience. Reviewing is, arguably, the most effective way of keeping abreast of developments and research in our community of practice, although it can be resource intensive. The review process for MedEdPublish is a little different to reviewing for more traditional medical journals, so here is some guidance. Although the guidance is particularly suited to MedEdPublish, it is likely to be suitable for other medical and health professional education journals. Further, the content may be of interest to potential authors, as it describes features that may receive better reviews.

## Performing the review

The time required is about 30-60 minutes per manuscript. If the topic is in your area of expertise, writing a review may be quicker, although it may be tempting to offer too much advice, or perhaps even re-write the paper! The potential for conflict of interest should, if anything, be over-declared, allowing the editors and readers to decide. The medical education community is small, so any links through institutions, committees and research interests are possible conflicts. If not in your area of expertise, a ‘generalist’ view is still often very useful, but this should be stated in the review. Similarly, reviews by the general readership, particularly students and recent graduates, are welcome, but this background should be stated to provide the context of your perspective. For less experienced reviewers, a systematic approach to the analysis of a paper is helpful. One evidence-based-medicine textbook that has excellent checklists for the appraisal of research papers (not just medical education) is ‘How to Read a Paper’ ( Greenhalgh, 2010).

It is worth asking the question: is there a difference between reviews and comments? The answer is yes, although there may be some overlap. Comments may be as short as a few words or a sentence and about only a single issue, whereas reviews should be a more detailed, comprehensive and structured analysis, with a combination of positive feedback and suggestions for improvement. Different kinds of papers may warrant different responses. For example, personal opinion manuscripts, editorials and commentaries may better suit comments rather than reviews. Reviews are welcome on all categories of manuscripts, but are essential for those presenting research, evaluation or new educational methods, where data are analysed and interpreted.

## Step 1. Read the manuscript through from beginning to end, then pause to consider it as a whole

**Scope.** Is the manuscript about medical education? Sometimes authors submit manuscripts that are more about health care or healthcare education of the public. While these may be interesting, they may fit better into other journals. Increasingly, some people submit out-of-scope manuscripts to either gain a publication falsely or to demonstrate that peer review is flawed. The editors will probably forward only those that are within scope, but sometimes this is a difficult judgement. Does this appear to be an original contribution, or have you read previously something either similar or based on the same data? ‘salami slicing’ refers to the dividing-up of data from a large study into two or more smaller studies, with same question and population described, without transparency, in more than one publication. This is not uncommon, particularly for studies involving large surveys. Reviewers should alert editors if this is suspected. Fraudulent research with fabricated data is rare in medical education, perhaps because this field is less likely to be a battlefield of grants, high citation index journals and institutional reputations, but reviewers should be mindful of the possibility. Is the manuscript reasonably current? Sometimes a paper is written or based on data collected several years ago, which may be of limited value now. If particular expertise may be helpful for highly specialized or rather obscure topics, suggest this in the review.

**Ethics approval.** This is essential for any manuscripts that report data collected from patients or learners, indeed any situation where there could be perceived to be a power imbalance or potential for harm to participants. Ethics approval details (Ethics Committee name and approval number) should be provided. Some medical education evaluations are exempt from ethics approval processes because they are linked to quality assurance projects, but these approvals may not cover external publication. Evidence of exemption should be provided, along with a statement that the principles of the Declaration of Helsinki were followed ( World Medical Association, 2013).

**Writing skills.** Does the text make sense? Is there a clear finding or message? Ideally, a manuscript ‘tells a story’, albeit in a concise, structured manner. There should be a logical flow of ideas. Is the grammar, language and syntax correct? These can be particular challenges for authors writing in a second or third language, but are not restricted to this group. Auto-correct functions are often used but these can be ‘false friends’ that do not always get things right. Is the length appropriate for the message? The art of clear, concise writing may not come naturally, as novice authors often write as they would speak. This can result in a discursive, lengthy manuscript that does not hold a reader’s interest. Longer manuscripts are harder to review and often attract fewer reviews. Some journals publish word limits for certain kinds of manuscripts. Qualitative research reports often require more space to present results and interpretation. Reviewers should not correct pedantically minor errors of spelling or grammar, but add a statement that there are several errors that should be fixed.

**Authorship.** Is it clear what each author contributed? Conventions vary, but ideally all authors should have contributed to most parts of the underlying project and resulting paper. Contributions should be stated.

## Step 2. Read the manuscript again, but this time more strategically and focus on sections

Comment where possible on each section and take notes.

**Title.** Does the title encapsulate the message? Some readers read further only if the title fits their interests, so these few words are important. For research and evaluation manuscripts, titles should define the study design. Better titles help readers choose which abstracts to read.

**Abstract.** Does the abstract summarise adequately the manuscript? Many readers will not read complete articles, so it is important that the purpose, methods and outcomes are clear. Structured abstracts make this easier for research papers.

**Introduction/background.** Does the introduction/background section provide sufficient context to ‘set the scene’? This section should explain where the author began the journey, why this study is necessary and why this manuscript should be read.

**Methods.** This section is likely to be present only in reports of research or evaluation papers, where it is essential. Are the methods clear, grounded in theory and appropriate for the question? Quantitative research includes many different methods. The two most common are quasi-experimental designs (where attempts are made to reduce the number of dependent variables) and non-parametric approaches (because interventions are often applied to small populations that do not reflect a ‘normal’ curve). If in any doubt, seek or recommend opinion from a statistician. Qualitative research also includes many methods, but is very useful for seeking understanding of complex issues. There should be sufficient separation between researchers and the information gathering or analysis to minimize the potential for perceived bias. If in any doubt, seek or recommend an opinion from a qualitative researcher. The term ‘mixed methods’ is currently popular, but this is a rather lazy term that needs to be justified by explanation of why each method was chosen to address each part of the usually complex question. Manuscripts are often mostly descriptive, without complex analyses, but are still of value in medical education as similar initiatives are often applied in very different contexts.

**Results.** Are the results clearly presented, at least initially free of interpretation? The facts should speak for themselves. Ideally, tables and figures should summarise and augment the text, but the text should clearly describe results without the tables and figures.There should not be more than three tables or figures, which should summarise the key findings. If more space is required to provide a more complete view of results, these can be added as an appendix.

**Interpretation and Discussion.** These sections are usually separate for quantitative studies and often combined for qualitative studies. Are the findings supported by the results? Over-interpretation is not uncommon, particularly for novice researchers. Not all quantitative designs can clearly attribute causation. Evaluations of educational activities are often low level (satisfaction) and short term (before and just after), where impacts can be measured more easily, but may not be worth reporting. Demonstrating longer term impact on practice is harder, but more valuable. Ideally, evaluation of impacts on health care practices and outcomes are attempted, but this can be very difficult in education research. While quantitative studies may produce results that may be applicable in other contexts, qualitative research is best at identifying influencing factors and enhancing understanding, but cannot attribute causation or association or be extrapolated easily to other contexts. Are there implications for other education programs? What research questions have been addressed and what further questions have been identified? Are recommendations for change in practice supported by the analysis? Descriptive manuscripts are just descriptions without much analysis or interpretation, but can still inform or inspire others. Manuscripts reporting ‘negative’ findings may be very important, as others can learn from knowing what did not work.

**Conclusion/summary.** All manuscripts should end with a ‘so what’ statement. For research and evaluation reports, this could be a single paragraph that summarises why the study was done, the findings and what they might mean. This can be similar to the abstract, only shorter and less focused on methods. This is not the right place for discussion or justification of results and their implications.

**References.** Are the references current, comprehensive and accurate? It is worthwhile checking briefly some references at random by pasting them into a search engine. While ‘seminal’ references may be older, most references should be from the last 5 years or so and authors should not self-cite too often. Citations of original research papers are better than other papers that cite (and perhaps misinterpret) original research. Researchers sometimes observe that their research is cited to support something different from what the research found! Are any key references missing? Literature searches do not always identify every relevant paper; much depends on the searcher and the search engine.

**Limitations.** Are limitations adequately presented and discussed? Small studies may not have sufficient scale, sampling may have been restricted or unrepresentative, or specific contextual factors may affect validity; these should be mentioned.

## Step 3. Write the review

Most journals provide a framework with a series of text boxes to complete. MedEdPublish does not, allowing more freedom. It is safer to write the review in a word processing program, save it and then cut and paste into boxes, as directly typed text may be lost if there is a software glitch. Reviews should be polite, constructive and helpful. They are visible to all readers, as is the name of the reviewer. Because of the openness, it is best to think of the review as advice to a colleague. For each section, think of things that have been done well and things that could be done to make this a better manuscript if revised. The ‘feedback sandwich’ concept is relevant here ( Cantillon & Sargeant, 2008). The aim is to help authors and readers learn from the experience of publishing. Sometimes reviewers and authors correspond within the MedEdPublish website, creating a discussion thread that all readers can observe, enjoy and learn from. It is important to state what changes would earn a higher star rating, because the same reviewer is often asked, and should agree, to review a revision.

## Step 4. Provide the star rating

MedEdPublish and some other journals have a star rating. This is an overall, ‘global’ rating that takes everything into account, including relevance, interest and practicality, as well as methodological issues. The MedEdPublish website describes the attributes for each level and this should be followed.

## Summary

This article provides a guidance for conducting reviews of medical education manuscripts, with a focus on reports of research, evaluations and innovations. More concise comments, rather than formal reviews, may be more appropriate for other types of articles. Both reviews and comments can generate interactive discussion threads that contribute to learning. With the growth in submitted manuscripts and medical education journals, many more reviewers and reviews are needed to support the medical education community of practice. For authors, there may also be key messages for how to write a better manuscript on medical and health professions education.

## Take Home Messages

• Reviewing medical and health professional education manuscripts is similar to reviewing in other fields, but there are some differences.
• Reviewing is an excellent way of keeping abreast of developments.
• The open discussion threads can provide valuable learning for authors, reviewers and readers.
• Potential authors may gain some tips for writing better papers.

## Notes On Contributors

Richard Hays is the Editor of MedEdPublish. ORCID: https://orcid.org/0000-0002-3875-3134

Barbara Jennings is an Associate Editor of MedEdPublish. ORCID: https://orcid.org/0000-0003-3792-9182

Trevor Gibbs is an Associate Editor of MedEdPublish. ORCID: https://orcid.org/0000-0002-1776-6518

## Declarations

The authors are all Editors of MedEdPublish.
