# Supplementary material for: How to review a paper on medical education
Source: MedEdPublish (2016). 2019 Jul 29;8:158. [Version 1] doi: 10.15694/mep.2019.000158.1 (PMC10712492; doi:10.15694/mep.2019.000158.1)
Supplement: Peer review history

# Reviewer report: Reviewer response for version 1

**DOI:** 10.21956/mep.19903.r30134

**Published:** 2020-01-25

**Authors:** Carla Todaro

**Affiliations:** 1 Azienda Sanitaria Universitaria Integrata di Trieste

This review has been migrated. The reviewer awarded 5 stars out of 5 This article represents a must to read for all the novice reviewers and for the researchers.This "handly guide" is easy to read, the key concepts are expressed in a systematic way and all the tips given for the review process are certainly helpful to make constructive reviews.

Reviewer Expertise:

NA

No decision status is available

# Reviewer report: Reviewer response for version 1

**DOI:** 10.21956/mep.19903.r30135

**Published:** 2019-08-17

**Authors:** Daniel Coyle

**Affiliations:** 1 NUI Galway

This review has been migrated. The reviewer awarded 4 stars out of 5 This article is a fresh take on the importance of reviewing medical education papers in medical literature. The abstract delivers an excellent and straight forward overview of the papers main information. The introduction contains concise background information for reviewers looking to improve or awaken their research reviewing skills. It provides great context for the main body of the paper. The major objective of the article is clear in the "Take Home Messages" section.The step by step guide outlined is very useful. The inclusion of ethics in the reviewing process is particularly relevant and important in the medical world today. The main point the authors make is that reviewers should try to target every aspect of their articles. With an ever-growing surplus of medical education papers I will certainly be implementing the above mentioned tips and suggestions in future reviewing activity.

Reviewer Expertise:

NA

No decision status is available

# Reviewer report: Reviewer response for version 1

**DOI:** 10.21956/mep.19903.r30133

**Published:** 2019-08-13

**Authors:** Olle ten Cate

**Affiliations:** 1 UMC Utrecht

This review has been migrated. The reviewer awarded 4 stars out of 5 • This is in interesting contribution to the field, reminding about something that the Dutch call "Droste-effect": a Droste cocoa box showing a nurse carrying a tray with hot chocolate and a Droste cocoa box showing a nurse carrying a tray with hot chocolate and a Droste cocoa box showing... etcetera. This is a paper to be reviewed instructs potential reviewers how to review a paper. It merges a journal article with instructions for reviewers for this same journal in which it is published soliciting post publication reviews.• For inexperienced reviewers is it a good guideline, but to be used flexibly, as everyone develops their own routines in reviewing. I personally like to print the paper and directly jot comments in the margins while doing the initial read (reflecting how a reader would experience the text), at variance with the authors' recommendation.• What I missed in this paper is how to respond to the first question that arises when one receives a review request, that is: Accept or decline? That leads to the consideration What is a reasonable number of papers to review? Rule of thumb I like is: review at least the same number of peer reviewed papers one published per year, times the average number of reviews a manuscript has (usually around 3), divided by the average number of co-authors of one's own papers. So, if you publish 4 papers per year, on average with 4 co-authors , a reasonable number of reviews to do per year is (4*3)/4 = 3 papers to review per year at least, i.e. every 3-4 months one review, to keep the system as a whole sustainable.• "Reviewers should not correct pedantically minor errors of spelling or grammar, but add a statement that there are several errors that should be fixed" - Hmm. I'm not sure. In fact those small suggestions are easily processed, and authors with less language proficiency are very pleased to be pointed at them. This touches a more fundamental question: is a review just meant to support an editorial decisions (then authors of this paper are right) or also to support the manuscript authors in revisions. I think both. • A smaller comment regards the expected sections of a manuscript to be reviewed. I would add that the Introduction should should end with a research question or equivalent.Two comments I have specifically regard reviewing for MedEpPublish.• Most journals help reviewers and responding authors by providing line numbering. Reviewing for MedEdPublish has the disadvantage that there are no line numbers to refer to in comments, not even page numbers• Not clear what to do if the reviewer judges that the paper should not be published. I recently reacted to a journal "I would be very concerned if this manuscript would be published". Fortunately the current manuscript is great and only requires minor revisions. I hope it will happen! Despite the fact that I did not follow all instructions for reviewers for this journal.

Reviewer Expertise:

NA

No decision status is available

# Reviewer report: Reviewer response for version 1

**DOI:** 10.21956/mep.19903.r30131

**Published:** 2019-08-08

**Authors:** Balakrishnan Nair

**Affiliations:** 1 Centre for Medical Professional Development and University of Newcastle

This review has been migrated. The reviewer awarded 4 stars out of 5 I found this article very useful. To check its utility , I just used this step by step process, to review an article for another journal and am glad to report it worked really well. As the authors mention, peer review is a challenging process because of the increasing number of journals and the demand on reviewers. Traditionally journals had a single or double blinded peer reviewing process with its own issues. .So the open process is very transparent and timely . This will avoid the power imbalance and lack of transparency This clear and brief paper will help new reviewers to get involved in the journal .

Reviewer Expertise:

NA

No decision status is available

# Reviewer report: Reviewer response for version 1

**DOI:** 10.21956/mep.19903.r30132

**Published:** 2019-08-08

**Authors:** Janie Dade Smith

**Affiliations:** 1 Bond University

This review has been migrated. The reviewer awarded 5 stars out of 5 I found this paper really useful in preparing a paper for publication and recommend it to others. It is clearly written, informative and helpful.

Reviewer Expertise:

NA

No decision status is available
